# Supplementary material for: Chemosensitization of prostate cancer stem cells in mice by angiogenin and plexin-B2 inhibitors
Source: Commun Biol. 2020 Jan 15;3:26. doi: 10.1038/s42003-020-0750-6 (PMC6962460; doi:10.1038/s42003-020-0750-6)
Supplement: Supplementary file 1 — Supplementary Information [file 42003_2020_750_MOESM1_ESM.pdf]

Supplementary Figure 1

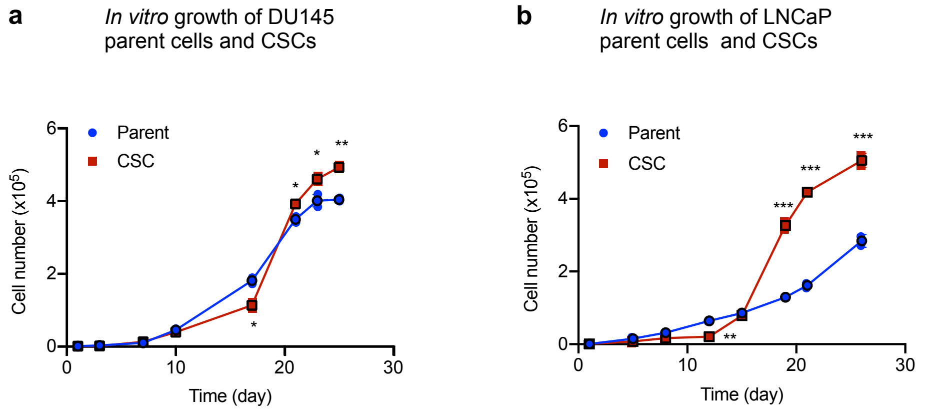

Supplementary Fig. 1 Growth characteristics of CSCs of DU145 and LNCaP. *In vitro* growth curve of CSCs and respective parent cells of DU145 (**a**) and LNCaP (**b**) were determined by MTS assay. Seeding density was 10,000 cells per well of 48-well plates (n=3). $^{*}p < 0.05$; $^{**}p < 0.01$; $^{***}p < 0.001$.

Supplementary Figure 2

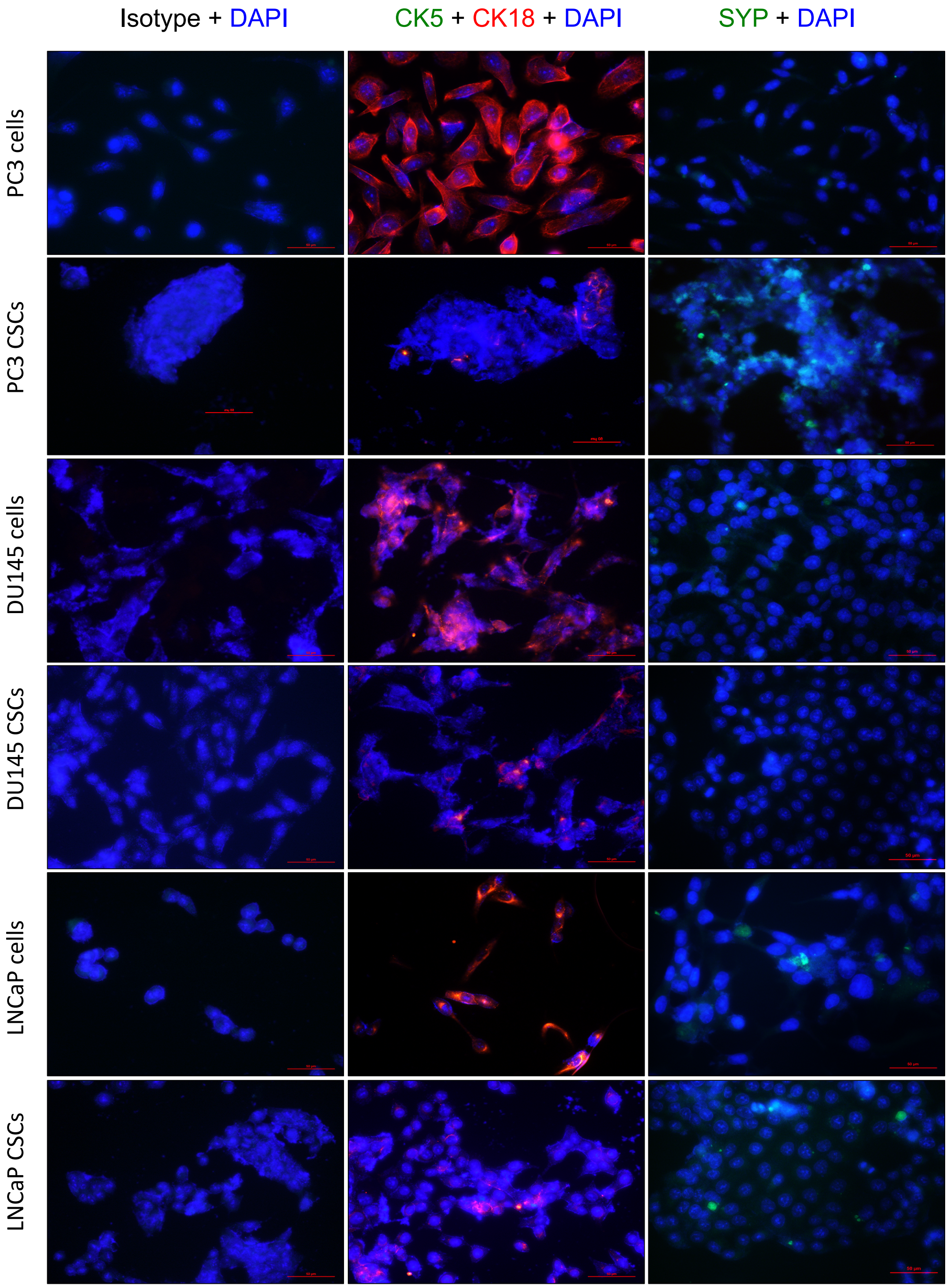

Supplementary Fig. 2 Immunofluorescence of basal, luminal, and neuroendocrine markers in prostate CSCs and parent cells. CSCs and parent cells were cultured on coverslips in DMEM + 10% FBS overnight, and stained for CK5, CK8, and SYP with specific antibodies.

Supplementary Figure 3

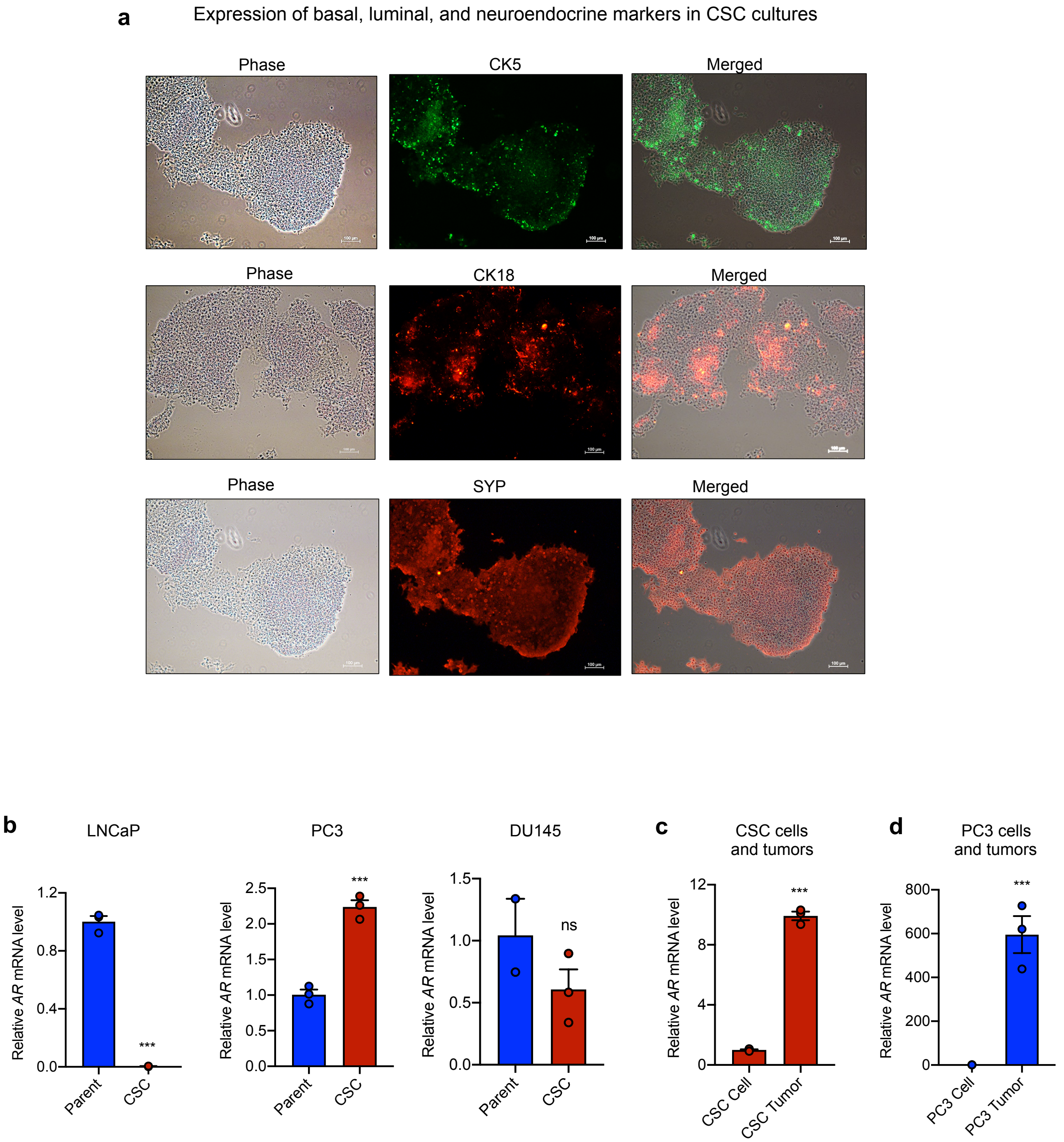

Supplementary Fig. 3 *In vitro* and *in vivo* differentiation of prostate CSCs. **a** CSC spheres of PC3 were seeded on cover slips and cultured in DMEM + 10% FSB for 4 days, and immunofluorescence staining was performed with specific antibodies. Scale bar: 100 µm. **b** Relative *AR* levels between prostate CSCs and respective parent cells. mRNA levels were determined by qRT-PCR and normalized to parent cells (n=3). **c** Relative *AR* levels between CSCs and CSC-derived tumors (n=3). **d**, Relative *AR* levels between PC3 and PC3-derived tumors (n=3). $^{***}P < 0.001$; ns, not significant.

Supplementary Figure 4

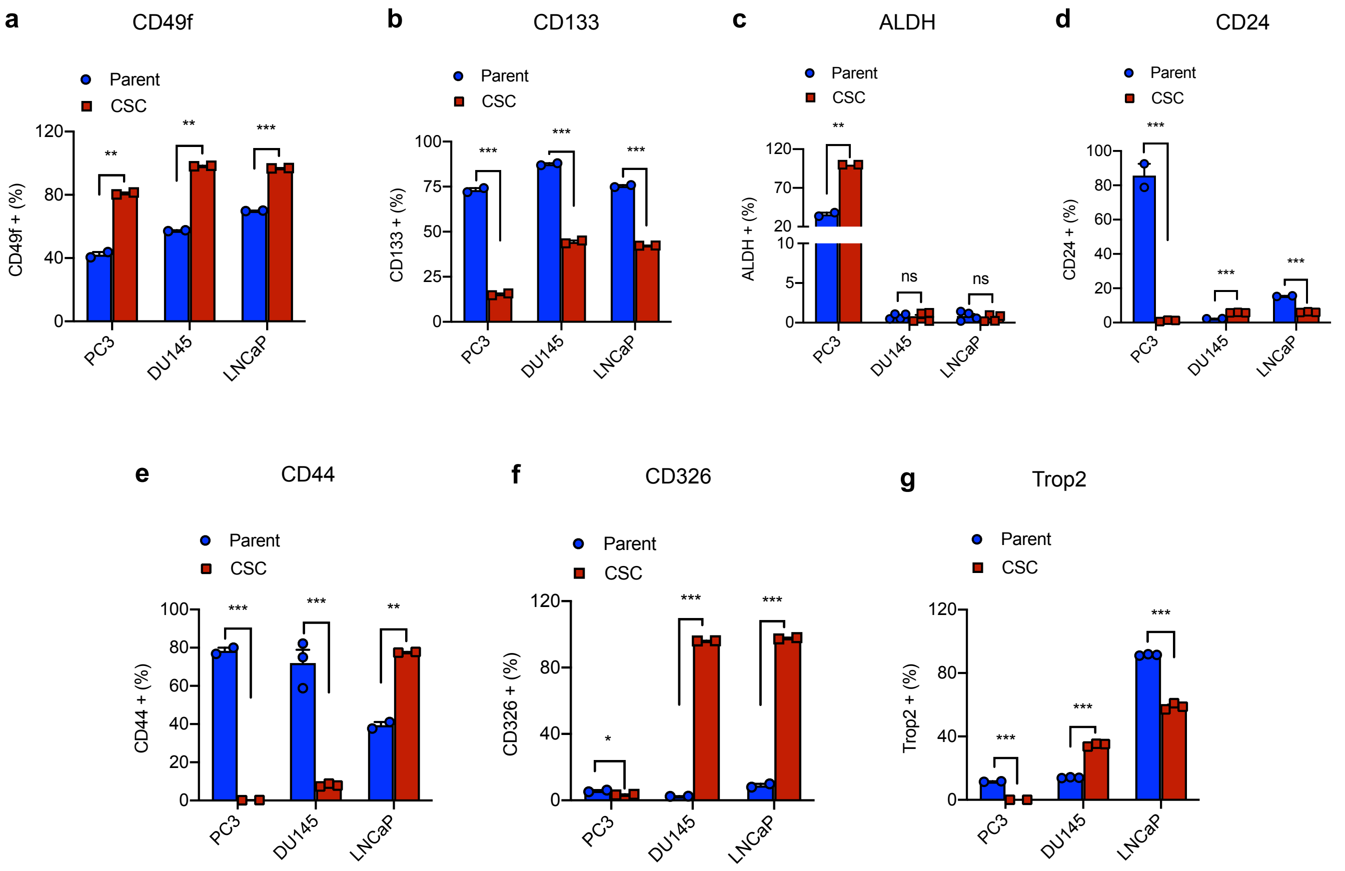

Supplementary Fig. 4 Expression of previously reported cell surface markers of prostate cancer stemness in CSCs and respective parent cells. Percentage of positive cells were determined by Flow cytometry analysis (n=4). $^{*}p < 0.05$; $^{**}p < 0.01$; $^{***}P < 0.001$; ns, not significant.

Supplementary Figure 5

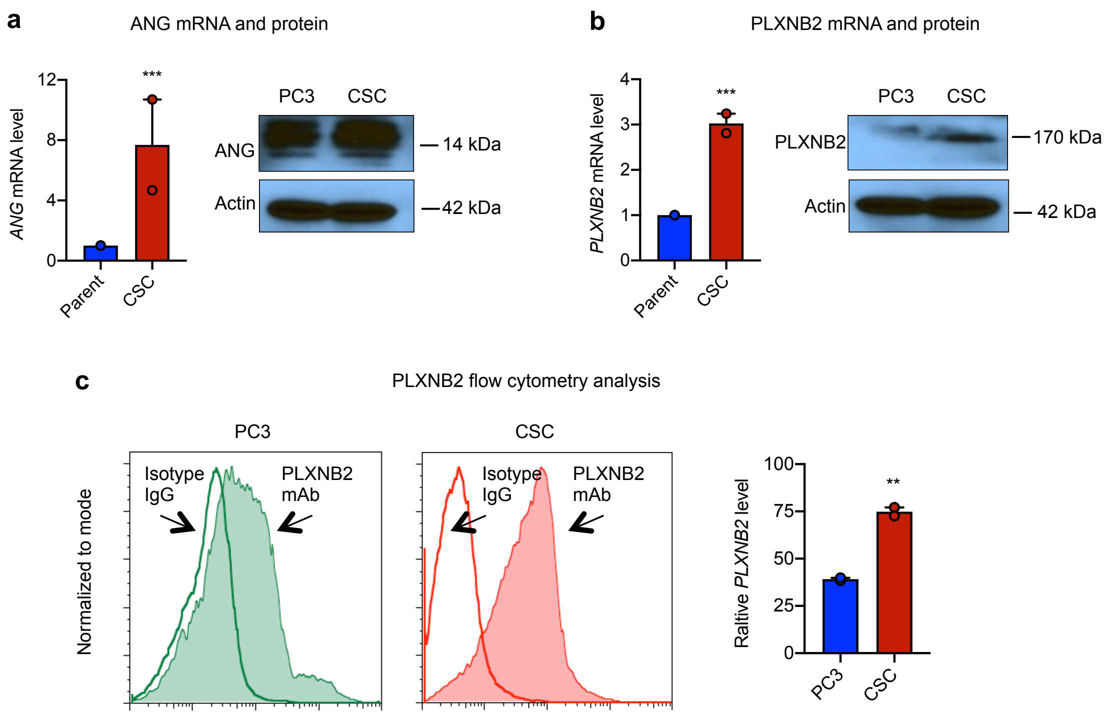

Supplementary Fig. 5 ANG and PLXNB2 levels in PC3 cells and CSCs. **a, b** mRNA and protein levels of ANG (**a**) and PLXNB2 (**b**). mRNA levels were determined by qRT-PCR and normalized to PC3 cells (n=3). Protein levels were determined by immunoblotting. **c** PLXNB2 protein level analyzed by flow cytometry with mAb17. Relative expression level was quantified by geometric mean, as calculated by FlowJo (n=3). $^{**}p < 0.01$; $^{***}p < 0.001$.

Supplementary Figure 6

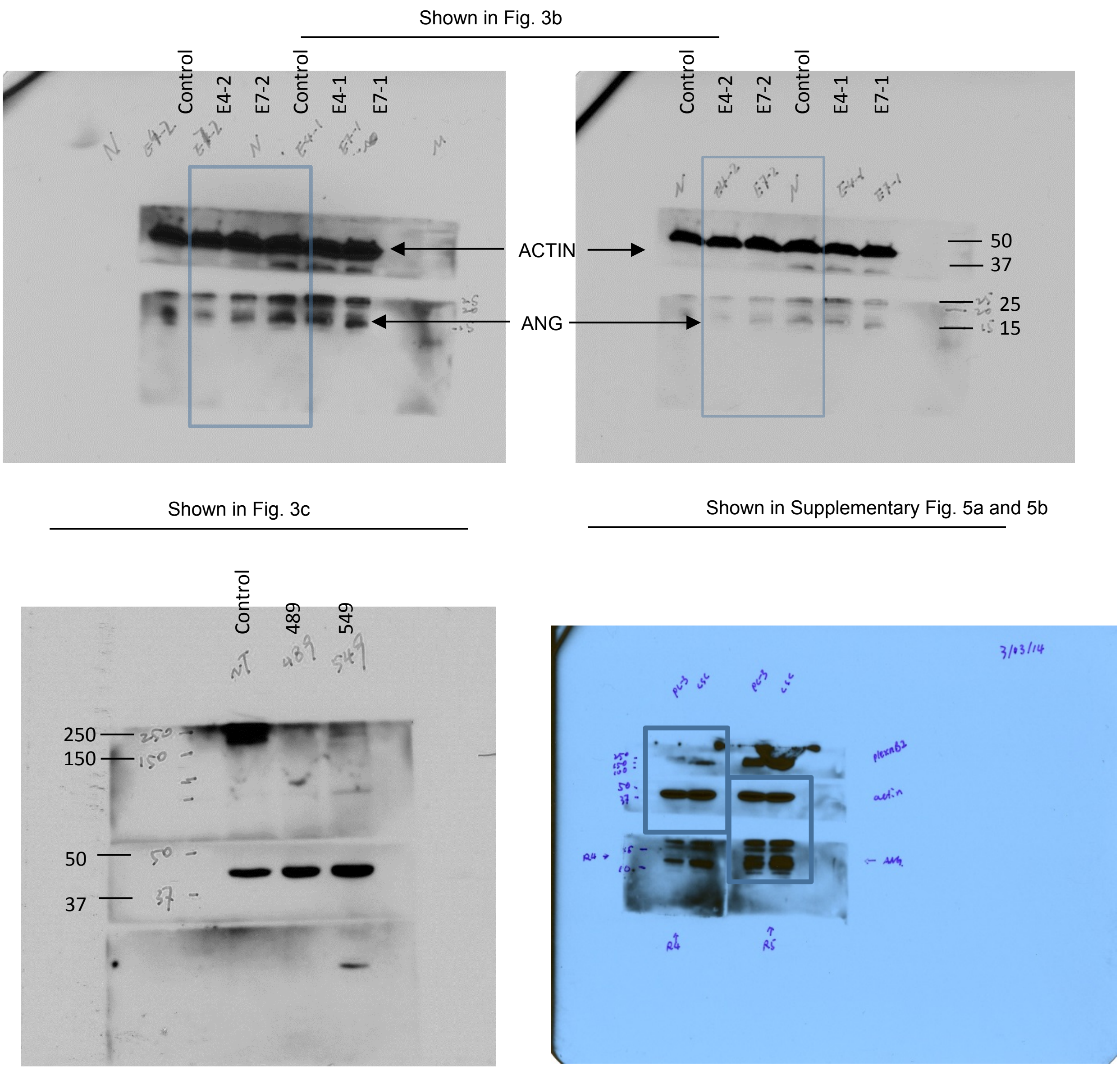

Supplementary Fig. 6 Uncropped immunoblots of ANG and PLXNB2 shown in Fig. 3b, 3c, and Supplementary Fig. 5a and 5b.

Supplementary Figure 7

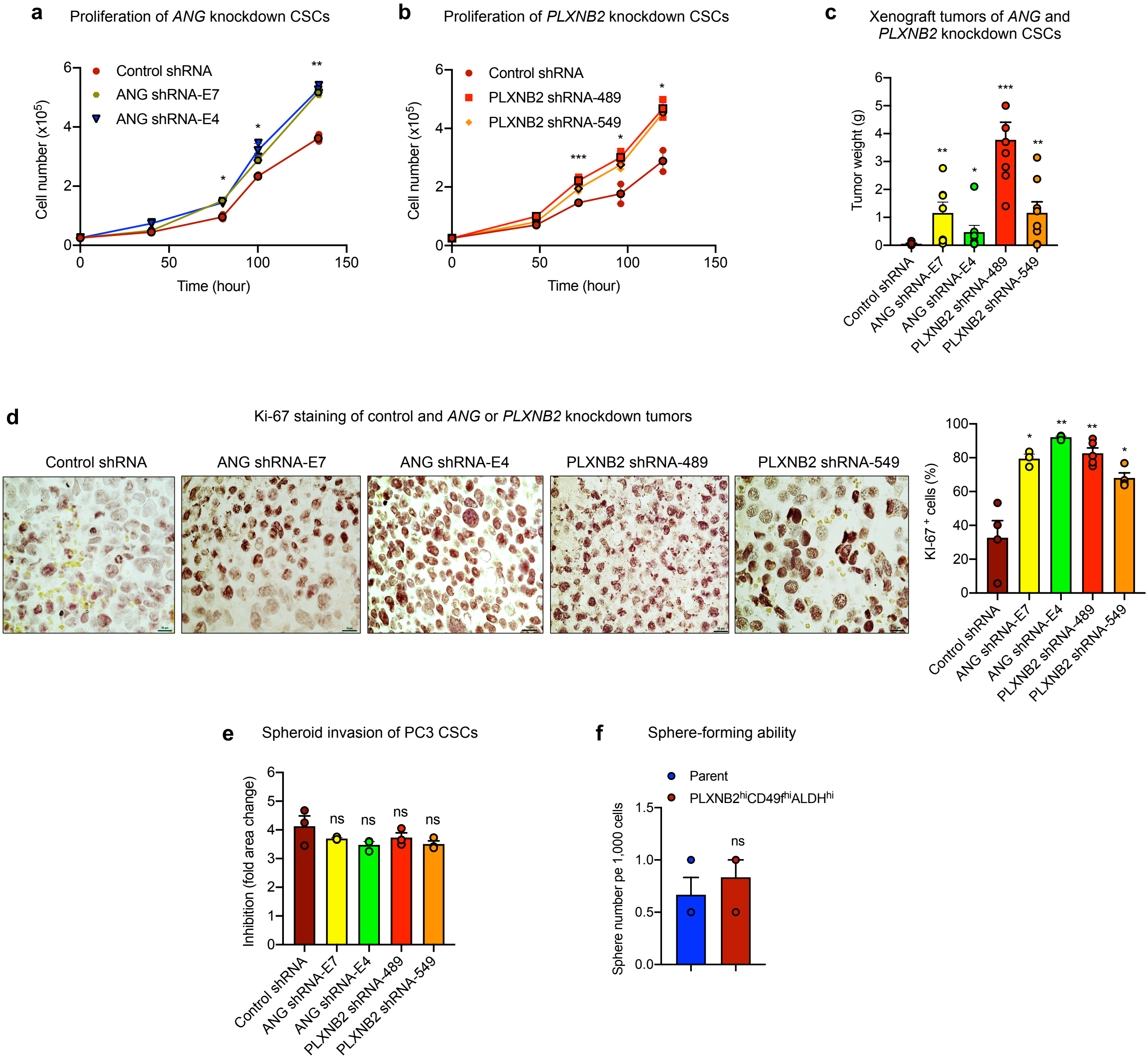

Supplementary Fig. 7 Effect of *ANG* and *PLXNB2* knockdown on CSC proliferation and tumor growth. **a, b** *In vitro* growth curves of *ANG* (**a**) and *PLXNB2* (**b**) knockdown CSCs. Cells were seeded in 24-well plates (15,000 per well) and cultured in DMEM + 10% FBS (n=3). **c** *In vivo* growth of *ANG* and *PLXNB2* knockdown CSCs. One hundred CSCs were subcutaneously inoculated into NSG mice, and xenograft tumors were excised and weighed 7 weeks post inoculation (n=7). **d** IHC of Ki-67 in xenograft tumors generated from control and *ANG* or *PLXNB2* knockdown CSCs. Scale bar: 10 µm. Percent of Ki67 positive cells were counted in 5 microscopic areas with a total cell number >1,000. **e** Spheroid invasion of control and *ANG* or *PLXNB2* knockdown CSCs. CSCs invaded into the matrix were recorded for 2 days. The invasive area was defined as that of the maximum cell dispersal. **f** Sphere formation of PC3 parent and sorted PLXNB2^{high}CD49f^{high}ALDH^{high} cells. $^{*}p < 0.05$; $^{**}p < 0.01$; $^{***}p < 0.001$; ns, not significant.

Supplementary Figure 8

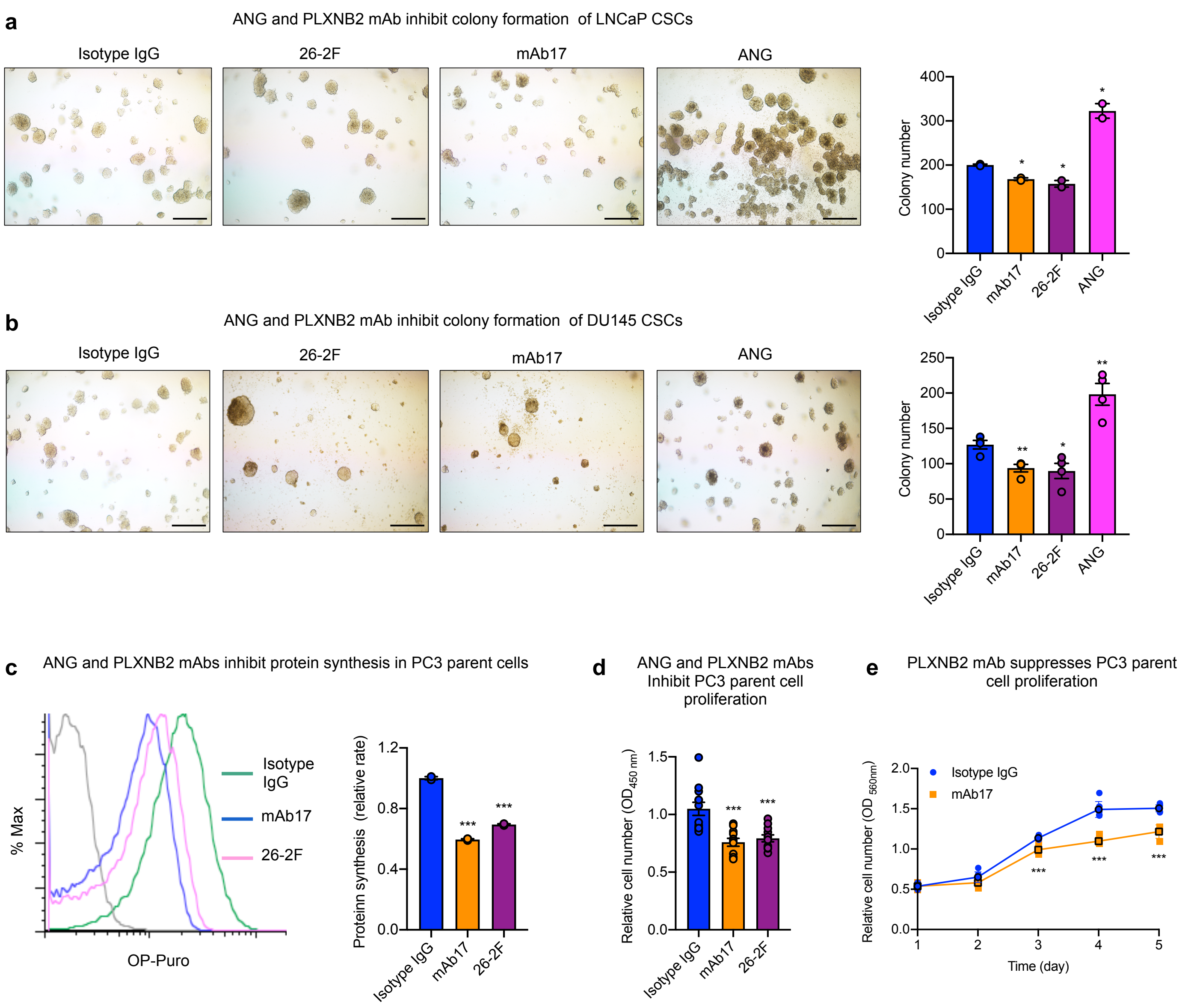

Supplementary Fig. 8 Effect of ANG and PLXNB2 mAbs on CSCs and parent cells. **a, b** Colony formation of CSCs from DU145 (**a**) and LNCaP (**b**) in the presence of 30 µg/ml isotype IgG, 26-2F or mAb17, 1 µg/ml ANG (n=3). Scale bar: 500 µm. **c** Protein synthesis in PC3 parent cells treated with 30 µg/ml of isotype IgG, 26-2F, or mAb17 for 24 h, determined by OP-Puro incorporation followed by flow cytometry. Geometric means were calculated and normalized to isotype IgG control (n=3). **d** Proliferation of PC3 parent cells in the presence of 30 µg/ml of isotype IgG, 26-2F, or mAb17. **e** *In vitro* proliferation of PC3 parent cells in the presence of 30 µg/ml isotype IgG or mAb17 (n=3). $^{*}p < 0.05$; $^{**}p < 0.01$, $^{***}p < 0.001$.

Supplementary Figure 9

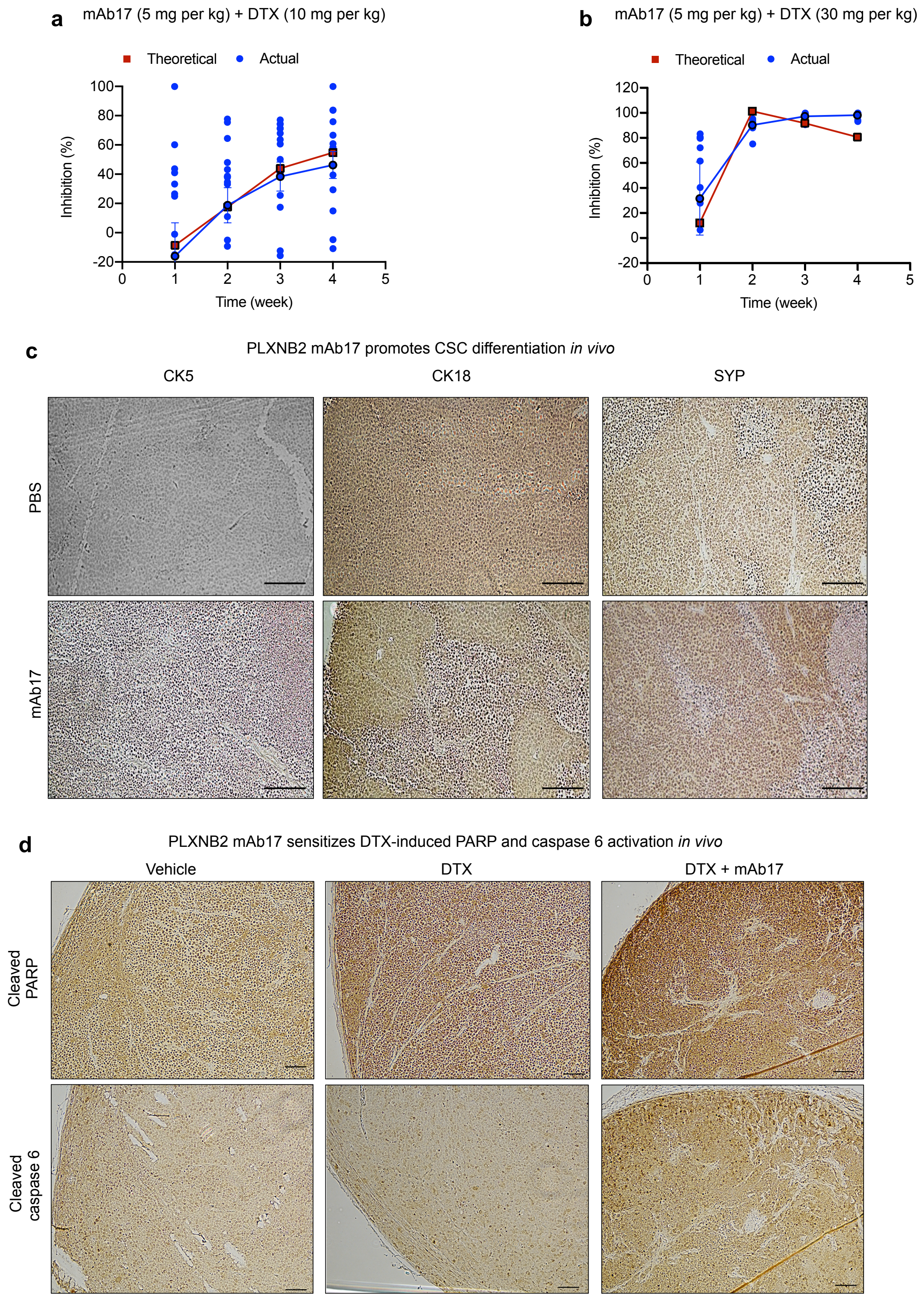

Supplementary Fig. 9 PLXNB2 mAb sensitizes CSC tumors to DTX. a, b Observed and theoretical inhibition of combinatorial treatment of 10 mg/kg DTX + 4.8 mg/kg mAb17 (**a**) or 30 mg/kg DTX + 4.8 mg/kg mAb17 (**b**) on CSC tumor growth. Theoretical values were calculated by adding the percent of inhibition observed in groups treated with mAb17 and DTX individually. Actual values were the percent of inhibition observed from the group treated with mAb and DTX together. **c** IHC staining of CK5, CK18, and SYP in CSC tumor tissues treated with PBS or mAb17 (n=3). Scale bar: 200 µm. **d** IHC of cleaved PARP and cleaved Caspase 6 in CSC tumor tissues from mice treated with PBS, 30 mg/kg DTX, and 30 mg/kg DTX + 4.8 mg/kg mAb17 (n=3). Scale bar: 100 µm.

Supplementary Figure 10

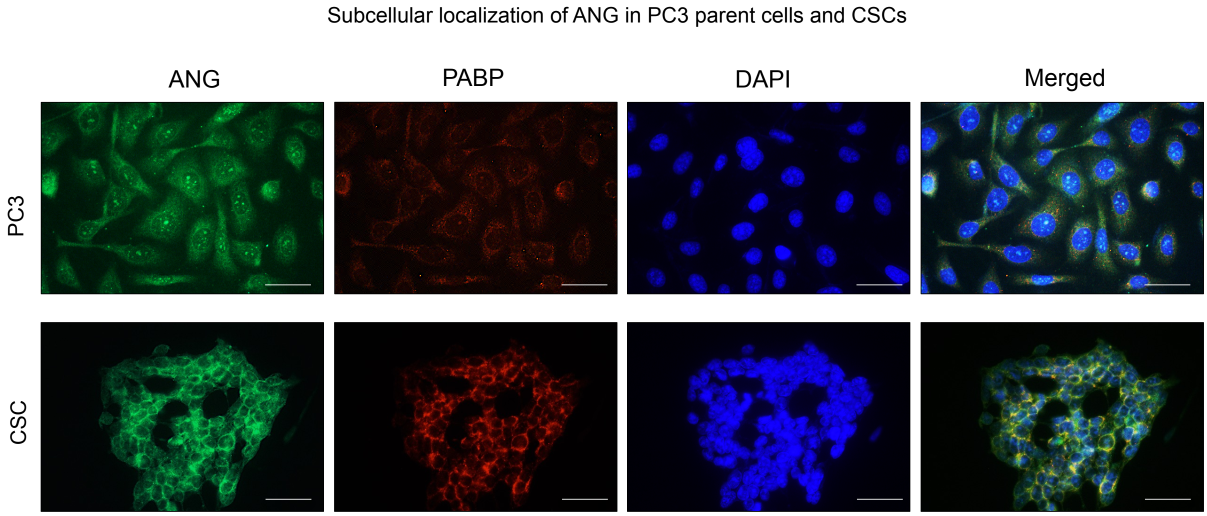

Supplementary Fig. 10 Stress granule localization of ANG in CSCs. Cells were seeded on coverslip, cultured in DMDM + 10% FBS overnight, fixed, and stained for ANG and PABP by immunofluorescence. Scale bar: 50 µm.

Supplementary Figure 11

Shown in Fig. 7a

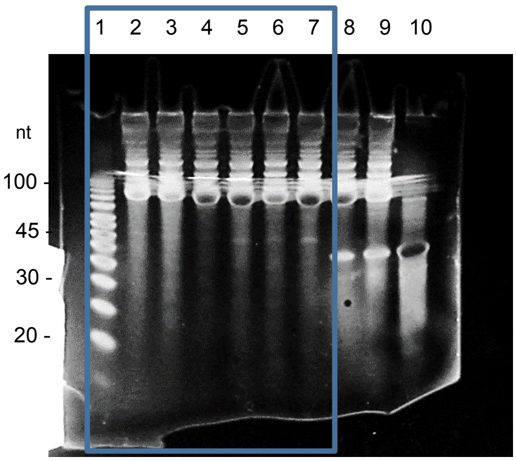

1. RNA ladder
2. PC3 Parent
3. PC3 CSC
4. DU145 Parent
5. DU145 CSC
6. LNCaP Parent
7. LNCaP CSC
8. Pure RNA
9. Pure RNA
10. Pure RNA

Shown in Fig. 7b

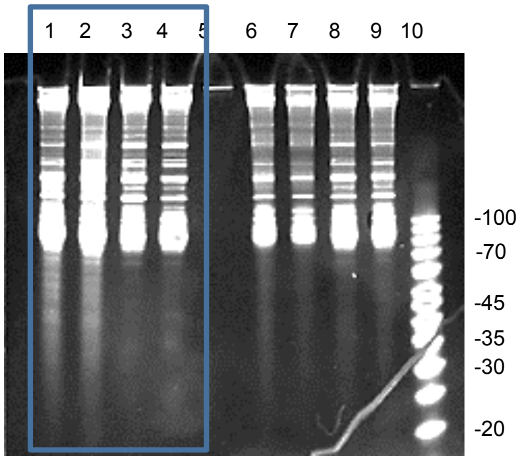

1. CSC PBS
2. CSC ANG
3. PC3 PBS
4. PB3 ANG
5. BLANK
6. CSC FBS
7. CSC FBS
8. PC3 FBS
9. PC3 FBS
10. RNA Ladder

Shown in Fig. 7c

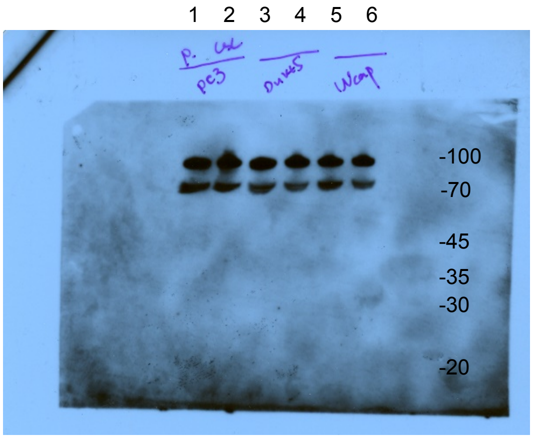

1. PC3 Parent
2. PC3 CSC
3. DU145 Parent
4. DU145 CSC
5. LNCaP Parent
6. LNCaP CSC

Shown in Fig. 7d

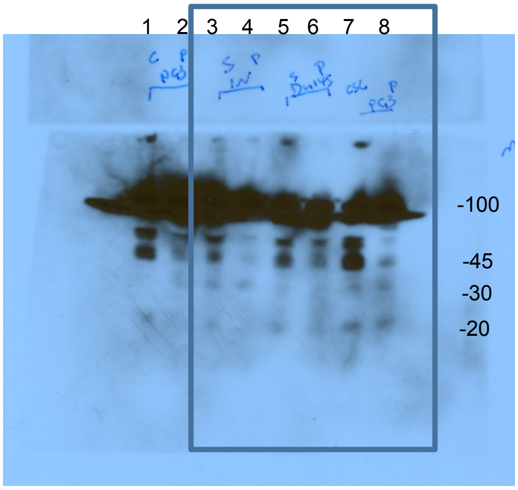

1. PC3 CSC
2. PC3 Parent
3. LNCaP CSC
4. LNCaP Parent
5. DU145 CSC
6. DU145 Parent
7. PC3 CSC
8. PC3 Parent

Shown in Fig. 7e

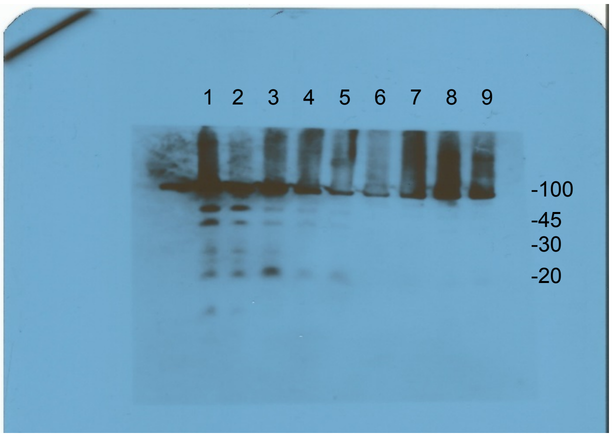

1. Control
2. PLXNB shRNA-489
3. PLXNB shRNA-549
4. ANG shRNA-E4
5. ANG shRNA-E7
6. Parent FBS control
7. Parent FBS control
8. Parent FBS control
9. Parent FBS control

Supplementary Fig. 11 Uncropped gels for small RNA and Northern blots of tiRNA and 3′-end fragment of of 5S rRNA shown in Fig. 7.

Supplementary Figure 12

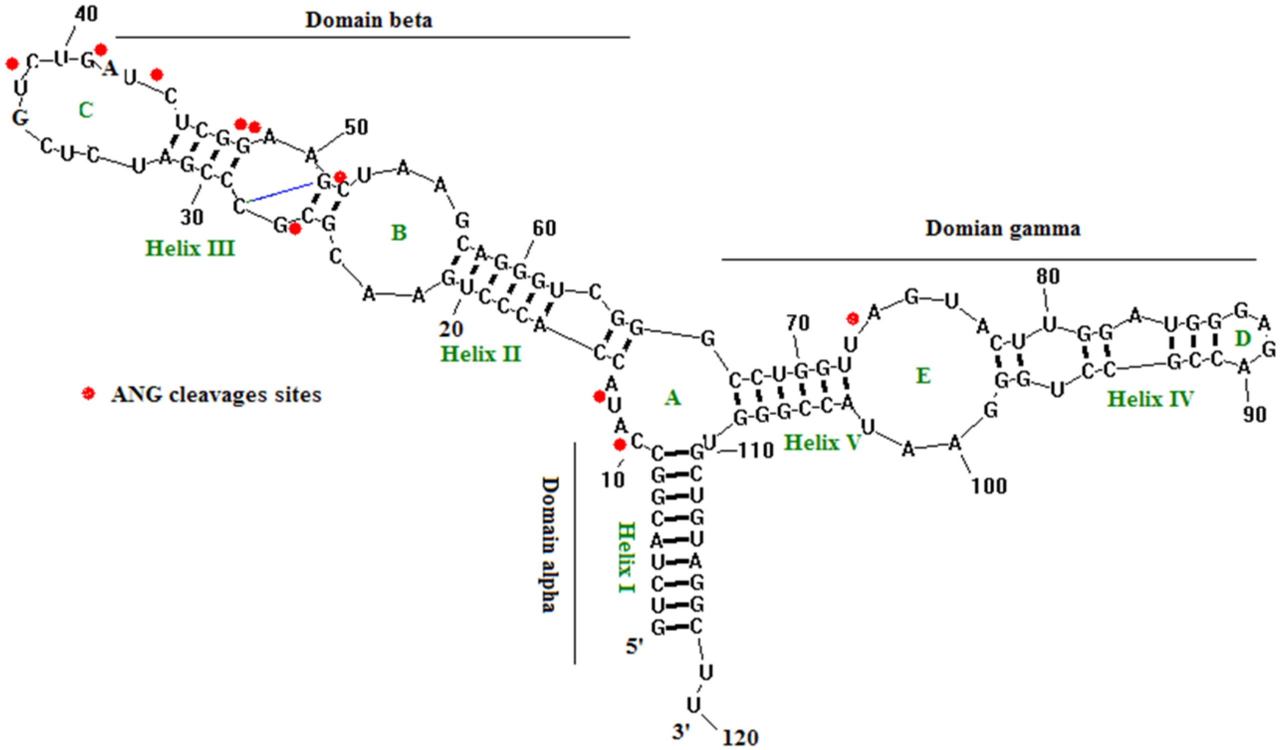

Supplementary Fig. 12 Secondary structure of 5S rRNA. ANG cleavage sites are denoted with red dots.

Supplementary Figure 13

Shown in Fig. 9a

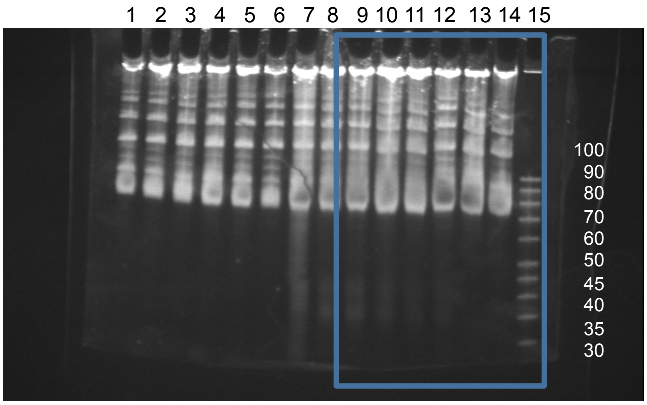

1-6. w/ FBS
7. HBSS 2 ug/ml ANG
8. HBSS 1.5 ug/ml ANG
9. HBSS 1.0 ug/ml ANG
10. HBSS 0.8 ug/ml ANG
11. HBSS 0.6 ug/ml ANG
12. HBSS 0.4 ug/ml ANG
13. HBSS 0.2 ug/ml ANG
14. HBSS 0 ug/ml ANG
15. RNA Ladder

Shown in Fig. 9b

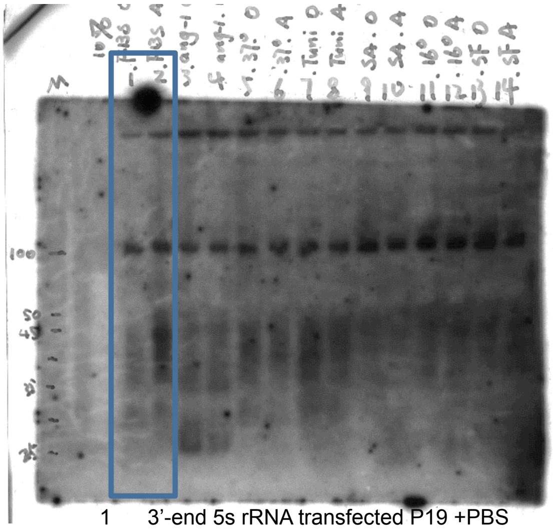

1 3'-end 5s rRNA transfected P19 +PBS
2 3'-end 5s rRNA transfected P19 + ANG
3-14. Other conditions w/o transfection

Shown in Fig. 9f (Bcl-2)

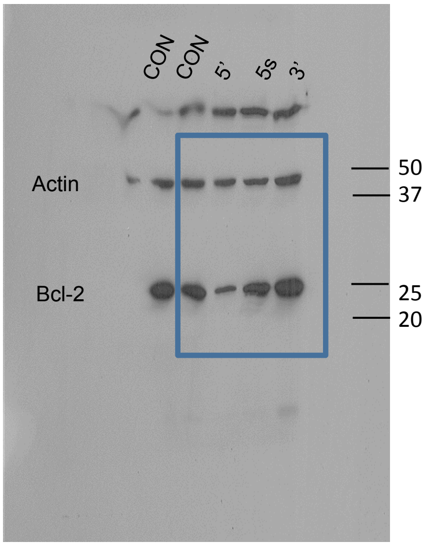

Shown in Fig. 9f (PARP)

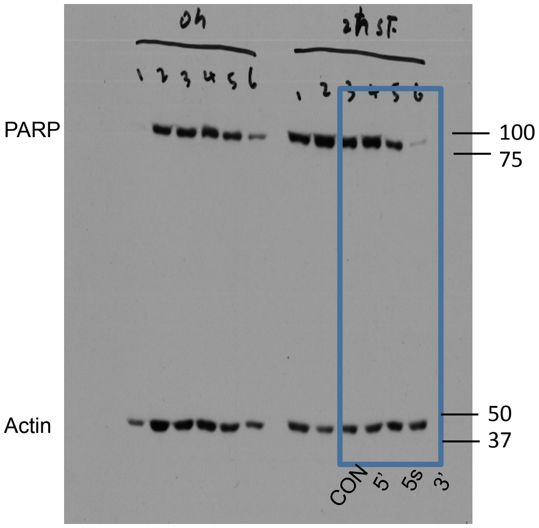

Shown in Fig. 9f (AIF)

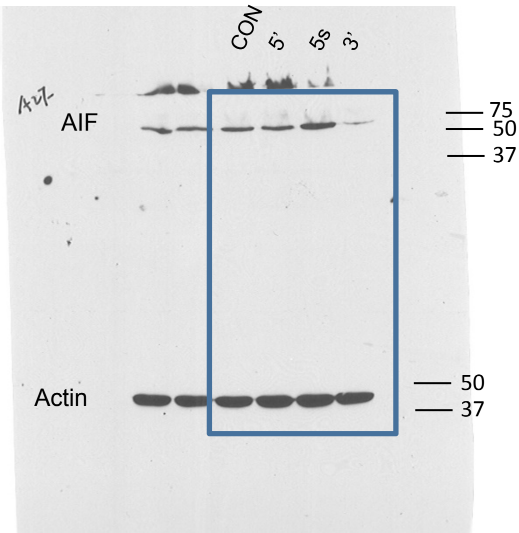

Supplementary Fig. 13 Uncropped gel of small RNA, Northern blot of 3'-end fragment of of 5S RNA, and immunoblots of Bcl-2, Parp-1, and AIF shown in Fig. 9.

Supplementary Table 1. Sequences of 5S rRNA fragments in CSCs

Clone No.	Occurrence	Sequences (5' to 3')
1-9	1	ACCACCCTGAACGCGCCCGATCTCGTCTGATCTCGGAAGCT
1-17	1	ATACCACCCTGAACGCGCCCGATCTCGT
1-20	1	GTCTACGGCCATACCACCCTGAACGCGCCCGATCTCGTCTGATCTCGGAAG
1-41	2	CTCGGAAGCTAAGCAGGGTCGGGCCTGGTT
1-43	1	ATACCACCCTGAACGCGCCCGATCTCGTCTG
1-47	1	GTCTACGGCCATACCACCCTGAACGCGCCCGATCTCGTCTGAT
2-1	3	GTCTACGGCCATACCACCCTGAACGCGCCCGATCTCGTCTG
2-2	4	GTCTACGGCCATACCACCCTGAACGCGCCCGATCTCGTCTGATCTCGG
2-4	2	GTCTACGGCCATACCACCCTGAACGCGCCCGATCTCGTCTGATCTCG
3-83	1	GTCTACGGCCATACCACCCTGAACGCGCCCGATCTCGTCTG
3-81	2	GTCTACGGCCATACCACCCTGAACGCGCCCGATCTCGTCTGATCTCGG
3-15	1	GTCTACGGCCATACCACCCTGAACGCGCCCGATCTCGTCTGATCTCG
3-11	1	ATACCACCCTGAACGC

Supplementary Table 2. Sequences of qRT-PCR primers

Genes	Forward (5’ to 3’)	Reverse (5’ to 3’)
CK5	CAAGGTTGATGCACTGATGG	TCAGCGATGATGCTATCCAG
CK8	AATGAATGGGGTGAGCTGGAG	CCTGATGGACATGGTAGAGGC
CK14	GGCCTGCTGAGATCAAAGAC	GTCCACTGTGGCTGTGAGAA
CK18	GAGATCGAGGCTCTCAAGGA	CTGAGATTTGGGGGCATCTA
CK19	TTTGAGACGGAACAGGCTCT	AGCTCTTCCTTCAGGCCTTC
GSTP1	GAGGACCTCCGCTGCAAATA	CAGCAGGGTCTCAAAAGGCT
p63	CCTGACCCTTACATCCAGCG	CTCTGGGACATGGTGGATCG
CHGA	GTCGGGGTATATAAGCGGGG	CGTCTGTCGGTCGATCCTC
SYP	TTGTGCCAACAAGACCGAGA	GCCTGAAGGGGTACTCGAAC
GAPDH	GGAAGGTGAAGGTCGGAGTCA	GTCATTGATGGCAACAATATCCACT
GPI	AGGCTGCTGCCACATAAGGT	CCAAGGCTCCAAGCATGAAT
CLDN4	GAGAAACTGGTCAGGAGGA	ACCGTGAGTCAGGAGATAAA
SOX2	GAGAGAAAGAAGAGGAGAGAGA	GCCGCCGATGATTGTTAT
KLF4	CGCCTTGCTGATTGTCTATT	GCCGAGATCCTTCTTCTTTG
BMP7	CTAACCAAGTGTCCCGATTT	GAGGCTGAGTGCATACTATTT
MUC1	GGAGACACAGTTCAATCAGTAT	TGGGCAGAGAAAGGAAATG
ITGA2	CTGTGGCTGTCTTGTTTCT	CCAAATCATCTCAGGATCTACC
DNMT1	CTACTTCCTCGAGGCCTATAA	TGCCCTTCCCTTTGTTTC
Cyclin D1	AGCTCCTGTGCTGCGAAGTGGAAAC	AGTGTTCAATGAAATCGTGCGGGGT
GATA3	ACCACAACCACACTCTGGAGGA	TCGGTTTCTGGTCTGGA GCCT
BCL-2	GTGGATGACTGAGTACCTGAAC	GCCAGGAGAAATCAAACAGAGG
BCL2L1	GCTACCGGGCCGATGAA	GATTCTGAAGGGAGAGAAAGAGC
XIAP	CCTGCAGGATTGCCTTCCTAA	TGATGTCTGCAGGTACACAAGT
BAX	GCCCTTTTGCTTCAGGGTTT	TGAGACACTCGCTCAGCTTC
BAK1	GCAGGCTGATCCCGTCC	CTGCGGAAAACCTCCTCTGT
BAD	CTTGGGCCCAGAGCATGT	TCTGCAGAGCTGGAGTCTTCC
TP53	CAGTCTTGAGCACATGGGAGG	GGCGACTGTCCAGCTTTGT
MYC	CGGTTTTCGGGGCTTTATCTAAC	AGTTTCGTGGATGCGGCAAG
5S rRNA	GGCCATACCACCCTGAACGC	CAGCACCCGGTATTCCCAGG
AR	TCGGAAAGGTCTGGTTGGTG	ACAGAACACAGGTGTGCCAA
PLXNB2	TGGTTCCTGCTGTAGCCATC	GATGTCTCCGTGCTTCCTGA
