# Supplementary material for: Chemosensitization of prostate cancer stem cells in mice by angiogenin and plexin-B2 inhibitors
Source: Commun Biol. 2020 Jan 15;3:26. doi: 10.1038/s42003-020-0750-6 (PMC6962460; doi:10.1038/s42003-020-0750-6)
Supplement: Supplementary file 2 — Description of additional supplementary files [file 42003_2020_750_MOESM2_ESM.docx]

**Description of additional supplementary files**

**Supplementary Data 1.** Source data used for graphs shown in Figure 1.

**Supplementary Data 2.** Source data used for graphs shown in Figure 2.

**Supplementary Data 3.** Source data used for graphs shown in Figure 3.

**Supplementary Data 4.** Source data used for graphs shown in Figure 4.

**Supplementary Data 5.** Source data used for graphs shown in Figure 5.

**Supplementary Data 6.** Source data used for graphs shown in Figure 6.

**Supplementary Data 7.** Source data used for graphs shown in Figure 7.

**Supplementary Data 8.** Source data used for graphs shown in Figure 8.

**Supplementary Data 9.** Source data used for graphs shown in Figure 9.

**Supplementary Data 10.** Source data used for graphs shown in Supplementary Figures 1, 3, 4, 5, 6, and 7.
