# Supplementary material for: Chemosensitization of prostate cancer stem cells in mice by angiogenin and plexin-B2 inhibitors
Source: Commun Biol. 2020 Jan 15;3:26. doi: 10.1038/s42003-020-0750-6 (PMC6962460; doi:10.1038/s42003-020-0750-6)
Supplement: Supplementary file 13 — Reporting Summary [file 42003_2020_750_MOESM13_ESM.pdf]

nature research

Corresponding author(s): Guo-fu Hu

Last updated by author(s): Dec 10, 2019

Reporting Summary

Nature Research wishes to improve the reproducibility of the work that we publish. This form provides structure for consistency and transparency in reporting. For further information on Nature Research policies, see Authors & Referees and the Editorial Policy Checklist.

Statistics

For all statistical analyses, confirm that the following items are present in the figure legend, table legend, main text, or Methods section.

n/a	Confirmed	
☐	☒	The exact sample size (*n*) for each experimental group/condition, given as a discrete number and unit of measurement
☐	☒	A statement on whether measurements were taken from distinct samples or whether the same sample was measured repeatedly
☐	☒	The statistical test(s) used AND whether they are one- or two-sided<br>*Only common tests should be described solely by name; describe more complex techniques in the Methods section.*
☒	☐	A description of all covariates tested
☐	☒	A description of any assumptions or corrections, such as tests of normality and adjustment for multiple comparisons
☐	☒	A full description of the statistical parameters including central tendency (e.g. means) or other basic estimates (e.g. regression coefficient) AND variation (e.g. standard deviation) or associated estimates of uncertainty (e.g. confidence intervals)
☒	☐	For null hypothesis testing, the test statistic (e.g. *F*, *t*, *r*) with confidence intervals, effect sizes, degrees of freedom and *P* value noted<br>*Give P values as exact values whenever suitable.*
☒	☐	For Bayesian analysis, information on the choice of priors and Markov chain Monte Carlo settings
☒	☐	For hierarchical and complex designs, identification of the appropriate level for tests and full reporting of outcomes
☒	☐	Estimates of effect sizes (e.g. Cohen's *d*, Pearson's *r*), indicating how they were calculated

Our web collection on statistics for biologists contains articles on many of the points above.

Software and code

Policy information about availability of computer code

Data collection	No commercial and custom code was used to collect data in this study.
Data analysis	Summit 4.3 and FlowJo software were used for FACS analysis. Rstudio was used to generate heatmap in qRT-PCR analysis. ImageJ was used for band density analysis in Western blots. SSPS v20 was used in two-way ANOVA analysis by Newman-Keuls multiple comparisons test.

For manuscripts utilizing custom algorithms or software that are central to the research but not yet described in published literature, software must be made available to editors/reviewers. We strongly encourage code deposition in a community repository (e.g. GitHub). See the Nature Research guidelines for submitting code & software for further information.

Data

Policy information about availability of data

All manuscripts must include a data availability statement. This statement should provide the following information, where applicable:

- Accession codes, unique identifiers, or web links for publicly available datasets
- A list of figures that have associated raw data
- A description of any restrictions on data availability

No datasets were generated or analyzed during the current study. All data generated or analyzed during this study are included in this published article and its supplementary information files.

Field-specific reporting

Please select the one below that is the best fit for your research. If you are not sure, read the appropriate sections before making your selection.

☒ Life sciences ☐ Behavioural & social sciences ☐ Ecological, evolutionary & environmental sciences

For a reference copy of the document with all sections, see nature.com/documents/nr-reporting-summary-flat.pdf

Life sciences study design

All studies must disclose on these points even when the disclosure is negative.

Sample size	No sample sizes were per-calculated. Biological and experimental replicates were determined empirically for particular experiments.
Data exclusions	No data was excluded from analysis
Replication	Reproducibility of experimental findings was verified by different investigator or by the same investigator at different time.
Randomization	Mice was randomly grouped in xenograft tumor experimts.
Blinding	The investigator who measured tumor sizes was blinded to the treatments.

Reporting for specific materials, systems and methods

We require information from authors about some types of materials, experimental systems and methods used in many studies. Here, indicate whether each material, system or method listed is relevant to your study. If you are not sure if a list item applies to your research, read the appropriate section before selecting a response.

Materials & experimental systems

n/a	Involved in the study	
☐	☒	Antibodies
☐	☒	Eukaryotic cell lines
☒	☐	Palaeontology
☐	☒	Animals and other organisms
☒	☐	Human research participants
☒	☐	Clinical data

Methods

n/a	Involved in the study	
☒	☐	ChIP-seq
☐	☒	Flow cytometry
☒	☐	MRI-based neuroimaging

Antibodies

Antibodies used	Monoclonal anti-human ANG (26-2F), monoclonal anti-human PLXNB2 (mAb17), and polyclonal anti-human ANG (R113) were prepared in house. No additives were used in these antibodies. Anti-actin was from Santa Cruz, Cat# sc-58673; Anti-tubulin was fromSanta Cruz, Cat# sc-73242; Anti-CD45.1-APC was from eBioscience, Cat# 17-0453-82; Anti-CD45.2-Pacific Blue was from Biolegend, Cat# 109819; Anti-Ki67-PE was from eBioscience, Cat# 12-5698-80; Anti-CD49f-PE was eBioscience, Cat#12-0495-83; Anti-CD133- PE was from Miltenyi Biotec, Cat#130-111-079; Anti-CD326-FITC was from Bio-Rad; Cat#MCA1870F; Anti-CD24 was from eBioscience, Cat# 14-0247-82; Anti-Trop2was from eBioscience, Cat# 14-6024-82; Goat F(ab')2 anti-Mouse IgG-PE was from eBioscience, Cat# 12-4010-82; Anti-CK18 was from Cell Signaling, Cat#4548; Anti-CK5 was from Novus Biologicals, Cat # NB110-56916; Anti-SYP was from Enzo Life Science, Cat #ADI-VAM-SV011-F; Anti-PABP was from Abcam, Cat# ab6125; Anti-mouse IgG (H+L), F(ab')2 Fragment (Alexa Fluor 555 conjugate) was from Invitrogen, Cat# A21425; Anti-rabbit IgG (H+L), F(ab')2 Fragment (Alexa Fluor® 488 conjugate) was from Invitrogen, Cat# A-11070; Anti-Bcl-2 (124) mouse mAb was from Cell Signaling, Cat# 15071; Anti-Bcl-2 (D17C4) rabbit mAb was from Cell Signaling, Cat# 3498; Anti-p53 was from Cell Signaling, Cat# 9282; Anti-cleaved Caspase-6 (Asp162) was from Cell Signaling, Cat# 9761; Anti-cleaved PARP (Asp214) (D64E10) rabbit mAb was from Cell Signaling, Cat# 5625; Anti-cleaved PARP (Asp214) (7C9) mouse mAb (Mouse Specific) was fromCell Signaling; Cat# 9548; Anti-Ki67 was from EMD Millipore, Cat# AB9260; Goat anti-Rabbit IgG (H + L) HRP Conjugate was from Bio Rad, Cat # 1706515; Goat anti-Mouse IgG (H + L) HRP Conjugate was from Bio Rad, Cat # 1706516; Anti-AIF was from Cell Signaling, Cat# 4642. Mouse CD45 APC-e780 was from eBioscience, Cat # 47-0451-82; Human CD45 Pacific Blue was from Biolegend, Cat # 304021;. Anti-hCD44 FITC was from MACS, Miltenyi Biotec, Cat#130-113-903; alkaline phosphatase-labeled anti-DIG IgG was from Roche, Cat # 11585762001).
Validation	Antibodies were validated with purified antigens if available, and with cell lysate by immunobtotting

Eukaryotic cell lines

Policy information about cell lines

Cell line source(s)	Human embryonic kidney HEK293 (Cat# CRL-1573), human prostate cancer PC-3 (Cat# CRL-1435), human prostate cancer LNCaP (Cat# CRL-1740), human prostate cancer DU145 (Cat# HTB-81), and mouse embryonal carcinoma P19 (Cat# CRL-1825) were from ATCC.
Authentication	PCR-based short tandem repeat (STR) profiling of 9 markers (Abmgood, Cat # C287) was performed annually and compared to ATCC datasheet to authenticate the identity of PC3, DU145, and LNCaP cells.
Mycoplasma contamination	Cells were screened for mycoplasma contamination every 3 months with e-Myco Mycoplasma PCR Detection Kit (Lilif, Cat # 25235)
Commonly misidentified lines (See ICLAC register)	None

Animals and other organisms

Policy information about studies involving animals; ARRIVE guidelines recommended for reporting animal research

Laboratory animals	NSG male mice (5-6 weeks of age) were purchased from the Jackson Laboratory, Stock# 005557.
Wild animals	None
Field-collected samples	None
Ethics oversight	All animal experiments were approved by Institutional Animal Care and Use Committee (IACUC) of Tufts University/Tufts Medical Center. All procedures were performed in accordance with protocols approved by IACUC of Tufts University/Tufts Medical Center.

Note that full information on the approval of the study protocol must also be provided in the manuscript.

Flow Cytometry

Plots

Confirm that:

☒ The axis labels state the marker and fluorochrome used (e.g. CD4-FITC).

☒ The axis scales are clearly visible. Include numbers along axes only for bottom left plot of group (a 'group' is an analysis of identical markers).

☒ All plots are contour plots with outliers or pseudocolor plots.

☒ A numerical value for number of cells or percentage (with statistics) is provided.

Methodology

Sample preparation	For cell cycle analysis, cells (1 x 10E7) were fixed and permeabilized using Cytofix/Cytoperm Fixation/Permeabilization Kit (BD) and stained with anti-human Ki67-PE (eBioscience, 1:20 in PBS + 2% FBS). DNA content was measured by 7-AAD. For stem cell marker analyses, cells, including CSCs and parent cells, were detached by trypsin-EDTA, washed with ice cold PBS, re-suspended in PBS containing 2% FBS at 1 x 10E6 cells per 150 µl, and stained with anti-hCD133-PE (Miltenyi Biotec) at dilution of 1:8, anti-hCD49f-PE (eBioscience) at 1:160, anti-hCD44-FITC (Miltenyi Biotec) at 1:100, anti-hCD326 FITC (Bio-Rad) at 1:100, on ice for 20 min in the dark. Stained cells were washed twice with PBS + 2% FBS at 350 x g and suspended in 300 µl of PBS + 2% FBS for analysis on a Cyan flow cytometer. For CD24 and Trop2, unconjugated primary antibodies (eBioscience) were used at 1:100 dilution, followed by PE conjugate of goat F(ab')2 anti-mouse IgG (eBioscience) at dilution of 1:125. PI or 7-AAD was used to exclude dead cells. For ALDH analysis, ALDEFLUORTM kit (Stemcell Technologies) was used. Briefly, 1 ml of 1 x 10E6 cells was mixed with 5 µl of activated ALDEFLUORTM reagent, half of the cells (0.5 ml) was immediately pipetted to control tube containing 5 µl of DEAB reagent, and both tubes were then incubated in a cell incubator for 45 min. Cells were then washed with ALDEFLUORTM buffer and centrifuged at 350 x g for 5 min. Cell pellets were suspended in 0.5 ml of ALDEFLUORTM buffer.
Instrument	Cyan, BD LSRII, FACSAria
Software	Summit 4.3 software and FlowJo software were used to collect and analyze flow cytometry data.
Cell population abundance	Cell population abundance was calculated based on the percent peak area of a particular population among the total area of all peaks.

Gating strategy	Using the FSC/SSC gating, debris was first removed by gating on the main cell population. For single color stained cells, isotype IgG control stained cells and mock treated cells were used to set up the boundaries between “positive” or “negative” staining populations. An identical positivity threshold was applied to all samples within a cell line.

☒ Tick this box to confirm that a figure exemplifying the gating strategy is provided in the Supplementary Information.
